# Advanced mechanical circulatory support for refractory cardiogenic shock after cardiac surgery: An eleven-year experience in Edinburgh

**DOI:** 10.1186/1749-8090-10-S1-A336

**Published:** 2015-12-16

**Authors:** Maziar Khorsandi, Kasra Shaikhrezai, Sai Prasad, Renzo Pessotto, William Walker, Edward Brackenbury, Geoffrey Berg, Vipin Zamvar

**Affiliations:** 1 Department of Cardio-Thoracic Surgery, Royal Infirmary of Edinburgh, Edinburgh, UK; 2 Department of Cardio-Thoracic Surgery, Golden Jubilee National Hospital, Glasgow, UK

## Background/Introduction

Post-cardiotomy cardiogenic shock (PCCS) occurs in 2-6% of patients undergoing surgical revascularization or valvular surgery. Approximately 0.5-1.5% of patients are refractory to maximal inotropic and intra-aortic balloon counter pulsation (IABP) support. Refractory PCCS leads to rapid multi-organ dysfunction syndrome and is an almost universally fatal clinical state without advanced mechanical circulatory support (AMCS) i.e. extra-corporeal membrane oxygenation (ECMO) or ventricular assist devices (VAD). However, the associated major complications and cost related to such complex devices has led to centralization of such valuable services to only a few UK centers.

## Aims/Objectives

We assessed the outcome of salvage AMCS for PCCS in a non-transplant cardiothoracic surgery unit over an eleven-year period.

## Method

The data was gained through the Royal Infirmary of Edinburgh cardiac surgery database. Our inclusion criteria included any patient from April 2004-April 2015 who had received salvage Veno-Arterial ECMO or VAD for PCCS (Cardiac Index < 2.2 L/min per square meter OR Systolic BP < 90 mmHg) refractory to IABP and maximal inotropic support following adult cardiac surgery.

## Results

We identified 16 patients who met the inclusion criteria in the aforementioned period. Age range was 34-83 years (Median 71). There was a large male predominance of 12 (75%). Overall 15 patients (94%) had received ECMO of which number, 12 (80%) had received central ECMO and 3 (20%) had received peripheral ECMO. 1 patient (6%) had VAD. Most common procedural related complication was haemorrhage. Massive stroke, Femoral artery pseudo-aneurysm, septic shock, and renal failure also occurred in this group. Overall survival was 31.2%. All survivors had NYHA class I-II on 24 months follow-up.

## Discussion/Conclusion

Our survival rate is identical to the reported data from previous studies. AMCS for refractory PCCS remains a controversial approach his is perhaps due to the high cost and serious complication rates. However the survivors appear to continue living with an acceptable quality of life.

